# The Work of Cultural Transition: An Emerging Model

**DOI:** 10.3389/fpsyg.2016.00427

**Published:** 2016-03-24

**Authors:** Tatiana V. Ryba, Natalia B. Stambulova, Noora J. Ronkainen

**Affiliations:** ^1^Department of Psychology, University of JyväskyläJyväskylä, Finland; ^2^Institute of Sports Science and Clinical Biomechanics, University of Southern DenmarkOdense, Denmark; ^3^School of Health and Welfare, Halmstad UniversityHalmstad, Sweden; ^4^Exercise, Health and Technology Center, Shanghai Jiao Tong UniversityShanghai, China

**Keywords:** athletic career, career construction, transitions, adaptability, migration, transnationalism, decent work

## Abstract

In today’s uncertain, fluid job market, transnational mobility has intensified. Though the concept of cultural transition is increasingly used in sport and career research, insight into the processes of how individuals produce their own development through work and relationships in shifting cultural patterns of meaning remains limited. The transnational industry of sports, in which athletes’ psychological adjustment to cultural transitions has implications for both performance and meaningful life, serves as a backdrop for this article. This study applied the life story method to interviews with 15 professional and semi-professional athletes, focusing particularly on the cultural transition aspect of their transnational athletic careers. The aims of the study were to identify the developmental tasks of cultural transitions and strategies/mechanisms through which cultural transitions were enacted. Three underlying mechanisms of the transition process that assisted athletic career adaptability were social repositioning, negotiation of cultural practices, and meaning reconstruction. Based on the data analyses, a temporal model of cultural transition is proposed. The results of this research provide professionals working in the fields of career counseling and migrant support with a content framework for enhancing migrant workers’ adaptabilities and psychological wellbeing.

## Introduction

In contemporary mobile society, relatively recent technological advances in transportation and communication have intensified people’s cross-border activities and experiences. The growing opportunities of linking migrant workers with transnational and local communities, as well as the diversity and fluidity of these ties, point to the emergence of new modes of transacting that require regular cultural transitions and sustained contact across national borders (Portes et al., 1999). The transnational field of sport industry arguably accentuates the new forms of cross-border exchanges, fostering a wider flow of athletes, coaches, sport tourists, artifacts, and value systems. The geographical movement of highly skilled amateur elite and professional athletes characterizes athletic career development today, which makes transnational mobility into a highly valuable commodity (Maguire and Falcous, 2011; Agergaard and Ryba, 2014). The production of mobility and adaptation to a changing context in cultural transition are crucial for initiating and maintaining the transnational career. That is, in addition to being able to establish mobility, it is important for athletic migrants to sustain their performance, which is often predicated on athletes’ adapt-abilities to create and maintain social relations and situated knowledge in the different localities they settle in, as well as those they leave behind. Although vocational psychology researchers have acknowledged that culture frames relational and working experiences, and the ways in which people respond to life events (e.g., Stead, 2004; Blustein, 2011), there seems to be a limited understanding of the processes activated in cultural transition that produce development through work and relationships in shifting cultural patterns of meaning.

As stated in the International Labour Organization (ILO) report that launched the concept of ‘decent work’ in 1999, the “rapid change in the global economy, engendering heightened competitive pressures, and reduced job security for many, has injected new uncertainties into the world of work” (International Labour Organization [ILO], 1999, p. 9). Roderick (2006, p. 261), for example, asserted that professional football/soccer players “exercise virtually no control over entry to the occupation, they have no monopoly over relevant bodies of knowledge, they cannot lay down standards of work or control the labor process.” Although each country has different deficits and needs in the decent work schema, the recent International Labour Organization (International Labour Organization [ILO], 2016) report further suggests that the concerns with social exclusion, employment quality, and security persist also at higher income levels. It has been estimated that each season one fifth of professional football players in the UK need to migrate to a new team nationally or internationally, or to find alternative work (Roderick, 2012). While professional and elite athletes’ mobilities cannot be compared to the experience of those confronted with involuntary migration due to war conflicts and extreme poverty, common themes including work insecurity and human dignity have emerged in previous studies with athletes (McGillivray et al., 2005; Roderick, 2006; Agergaard and Ryba, 2014). As indicated in literature on careers and migration, mobility necessarily involves perceptions of insecurity in terms of financial status, future career prospects, and inclusion and exclusion, which concern not only the lead migrants but also their spouses and families (Cohen et al., 2011). It has been reported that athletes’ conditions of work place profound emotional and economic constraints on their partners and spouses, who must cope with frequent geographical relocations, lack of stable social support network, and the partner’s frequent absence due to matches and competitions (Gmelch and San Antonio, 2001; Roderick, 2012; Ryba et al., 2015a). Injury and threat of de-selection from the team are additional career contingencies that athletes must navigate in order to sustain their status in the professional ranks. Studies indicate that many professional athletes compromise their health and continue playing despite pain and injury due to fear of de-selection, team pressure, guilt, or professional pride (Roderick et al., 2000; McGannon et al., 2013). Therefore, the concerns for decent work as outlined by the International Labour Organization (International Labour Organization [ILO], 1999, 2016), specifically in relation to job security, competitive pressures, and lack of social protection, are relevant also for careers in sport. Appropriating from this Research Topic’s call the notion of meaningfulness “in terms of cognitive, emotional, and relational domains of functioning,” the present research contributes to the debates on decent work by putting a spotlight on the relatively under-researched population of athletic migrants and the ways they construct meaning about work and lives in cultural transitions.

This article is based on the life stories of professional and semi-professional athletes whose athletic careers had been developed transnationally. Portes et al. (1999) distinguish between transnationalism ‘from above’ and ‘from below’ (see also Smith and Guarnizo, 2006). The highly successful athletes in high revenue sports construct their transnational careers predominantly through activities conducted by powerful sporting corporations, such as the National Hockey League (NHL) or the Association of Tennis Professionals (ATP) World Tour. However, most athletes’ transnational careers are the result of grass-roots activities, established channels of athletic migration, and initiatives facilitated by trans-local networks of their home country counterparts and friends of friends. Whilst both professional and semi-professional athletes engage in market work, there are considerable differences in the job arrangement between and within these groups. For methodological reasons, our analyses center on transnational athletes and their support networks; that is, the experiences of athletic migrants on a term-fixed contract/athletic scholarship typically based on performance outcomes, who travel extensively for training camps and competitions, maintain relationships and family life across national borders, and also develop relational ties within local communities.

## Cultural Mode of Being and Psychological Adjustment

This research is situated within cultural developmental psychology, which argues for the fundamentally cultural constitution of self in human development, and is informed by career development and cultural adaptation literature. Our theoretical starting point rests on the position developed by Vygotsky and Luria (1930/1993) that psychological processes are the *emergent* outcome of the transactions between an individual’s ontogenetic history in a particular sociocultural framework, and characteristics of the immediate tasks confronting the individual. Emphasizing historical development, cultural mediation and practical everyday activity, this approach considers individual traits as constructed by and also entwined with a specific medium of human development, which includes language, norms, customs, values, and artifacts (Vygotsky and Luria, 1930/1993; Stead, 2004; Markus and Kitayama, 2010). It is essential for our theorizing to understand the medium of human development as a set of symbolic resources, accumulated and transmitted across generations, which conveys its normative meanings in social practices and sociocultural institutions. One of the key symbolic resources of culture that is externalized in social interaction concerns the nature of the self and its relationship with others (Bruner, 1994; Adams and Markus, 2001). The acquired cultural patterns of interdependence between self and sociality is expressed in collaboratively constructed intersubjectivity—an implicit understanding “that is to some extent shared” (Moghaddam, 2010, p. 466). It is generally acknowledged that people with similar (sub)cultural background derive a common interpretation in social interactions (Locke, 2004).

In keeping with the aforementioned understanding, Kitayama et al. (2007) conceptualize self as a psychological system for behavioral regulation that is not merely an organization of conceptual schemas, but provides a principle for one’s spontaneous mobilization of thoughts, feelings, and actions, thus constituting the person’s mode of being. The mode of being is gradually formed in the dynamics of social interaction with significant adults (e.g., in the family, school, and sport) and is attuned to numerous characteristics of the surrounding physical and sociocultural environment. Because the self as a psychological system of self-regulation is generally maintained by intersubjectivity of shared norms in a given community, it is reasonable to anticipate cross-cultural variations in the ways of feeling, thinking, and acting in relation to oneself and others. The rupture of meanings and, indeed, of the cultural mode of being in transnational migration occurs because cultures and social contexts create diverse meaning systems as well as provide different opportunities and imperatives for developing and expressing individual psychosocial competencies, which form inner resources that individuals use to self-regulate and to direct their adaptive behaviors in psychological encounters with the environment (Savickas, 2005; Markus and Kitayama, 2010). Moreover, as Blustein (2001) and Blustein et al. (2004) assert, the ways individuals experience life aspirations, career motivations, and meaningful working life are rooted in relationships—thus, transforming vocational behavior into an inherently relational act.

Previous sport research provides the basis for suggesting that a cultural transition has important implications for migrant athletes’ adjustment to sport processes, such as playing style, team interaction, and coach–athlete relationship, and may create difficulties in their lives outside of sport (Duchesne et al., 2011; Schinke et al., 2013; Stambulova and Ryba, 2013; Ronkainen et al., 2014; Khomutova, 2015; Ryba et al., 2015b). Brandão and Vieira (2013), for example, indicated that Brazilian footballers’ poor adaptability hampers their professional careers inasmuch as 66% of 1029 players sent to foreign teams in 2010 returned to Brazil before completing their first season. Many players report inability to cope with loneliness and unfriendly climate, and to adapt to a new lifestyle. According to Schinke et al. (2013), who studied acculturation experiences of immigrant athletes and coaches in Canada, the key issues concern ‘navigating two world-views’ and ‘acculturation loads.’ The first theme refers to the ways in which immigrants navigate the shifting meanings of sport experiences in home and host countries. It also illustrates how the contextual contingency of meanings may present obstacles for migrating athletes as they seek to adapt within new cultural and sporting communities. The second theme highlights the salience of social environments in constituting acculturation either as shared with others (e.g., teammates and coaches) or as the sole responsibility of the migrated athlete to learn the norms and adapt to new contexts with no adjustment from the hosts. In a similar vein, Ryba et al. (2012) discussed acute cultural adaptation of Finnish female swimmers in their temporary migration to Australia as a process of negotiation of meanings between ‘here’ and ‘there,’ as well as of the extent to which athletes participated in daily social practices while developing a relationship with the new environment. The authors emphasized the constitutive role of culture in psychological adjustment to a cultural transition by demonstrating the ways in which team relatedness formed a discursive cultural space that provided security mediating the swimmers’ engagement with the Australian context. The latter argument resembles the proposition put forward by Blustein (2011, p. 11) that “culture functions as a form of holding environment for individuals” and “can serve as an essential relational resource as they cope with work-based transitions.”

## Rationale and Aims of the Present Study

The refocusing on the fluidity of adaptive processes and the emphasis on career adaptabilities in the vocational research literature is significant in revealing a socially constructed, relational nature of work-based experience (Blustein et al., 2004; Savickas et al., 2009; Schultheiss et al., 2011). Yet very few career studies have applied a transnational lens to migrant athletes’ life stories to discern the adaptive practices constituted within transnational mobility and career development. The transnational athletes’ intent for mobility in pursuit of professional and financial opportunities paired with often-strong identifications with professional qualifications and competencies, suggests that athletic migrants take an active role in designing their life through sport (Carter, 2011; Engh and Agergaard, 2013; Ryba et al., 2015a). This view is related to Bernaud’s (2014) observation that individual career choices are grounded in existential questions about the meaning that people give to their lives. With no intention to downplay agency, our previous research in this area has also conveyed an argument that athletes’ decision-making about the career and life course in general derives meaning in social interactions and take shape within a particular cultural and historical landscape that radically contextualizes the push and pull factors of migration (e.g., Stambulova and Ryba, 2014; Ryba et al., 2015b; Ronkainen et al., 2016). Taking into consideration the migrant workers’ need for adapting to a meaningful working life in transition, the question becomes how psychological tendencies for career adaptability are mobilized through daily practices in transient cultural contexts. The purpose of this study therefore was to understand what dynamics are particularly critical in cultural transition and what impact the time has on these processes. Our specific research aims were to identify the developmental tasks of cultural transition and basic psychological mechanisms underpinning the transition that assisted athletic career adaptability.

## Materials and Methods

The present study is situated in narrative psychology (Bruner, 1990; McAdams et al., 2001) and generally characterized as being exploratory. Our goal was to be sensitive to the diversity of individual experiences while, nevertheless, to analytically search for the basic psychological mechanisms underlying culturally patterned performativity. We have adopted life story interviewing as our methodological framework, which encourages participants to construct autobiographical narratives with minimal intervention from the researcher (Atkinson, 1998). Although life stories may be very different from one another, they reveal complex ways in which meanings are negotiated in the creation of a coherent life narrative as well as common social processes that have shaped people’s lives in similar circumstances (Bruner, 1994; Liversage, 2009). The benefits of a narrative approach for understanding career transitions, migration and displacement, vocational identity, and possible empowering of marginalized individuals have been widely acknowledged in vocational research (e.g., Cohen, 2006; Liversage, 2009; Schultheiss et al., 2011; Savickas, 2012). As Del Corso and Rehfuss (2011) suggested, the what, how, and why are inextricably connected through narrative. That is, *what* athletes do in career transition is based in part on their *why*, expressed in life-theme motives, and also *how*, which is indicative of their adaptive self-regulation processes. As career narrations provide a primary means of bringing meaning to sporting experiences, they can be analyzed for the psychological processes associated with transitions and patterns of career decisions (Carless and Douglas, 2009, 2013).

### Participants

Participants of this study were 15 professional and amateur elite (semi-professional) athletes in various sports. One athlete was Black, one athlete was mixed-race, and the rest were Caucasian. Four athletes were from ice hockey, two from basketball, two from soccer, two from handball, and one each from archery, orienteering, athletics, alpine skiing, and sport dance. Seven were male and eight were female, their ages ranged from 18 to 37 (median age was 26) and all of them experienced a cultural transition in association with various transnational mobilities. At the time of the interviews, two athletes were retired from competitive sport (aged 30 and 37) and one athlete (aged 26) considered herself as ‘temporally retired.’ Their educational background varied from high school dropouts to university graduates. The participants’ countries of origins included Bulgaria, Canada, Denmark, England, Estonia, Finland, Nigeria, Slovakia, and Sweden; all were fluent in the English language. For 13 athletes, athletic career development was a life-theme motive for migration. Two athletes’ transnational career was initiated by following a spouse/partner to another country, offering additional insights into the process of a cultural transition paired with an unassisted athletic career transition. Participants had numerous geographic migrations across and within different countries in the course of their transnational athletic careers, including the Nordic countries, UK, USA, Russia, Korea, China, and Australia. The diversity of transnational career pathways provided a rich comparative context for accentuating the psychosocial content and underpinning the processes animating cultural transition.

The participants were identified and contacted through personal networks. In selecting transnational athletes to interview, we followed Atkinson (1998, p. 27) suggestions to search for uniqueness and “someone who intrigues, inspires, fascinates, or perplexes you” in addition to being accessible. In line with ‘good’ interpretivist research, we relied not solely on the quantifiable prevalence of voices, but rather on those voices whose narrations captured something new and insightful in relation to investigated phenomena (Smith and Sparkes, 2009). Because the majority of the participants are easily identifiable in their countries of origin and current localities, we do not provide the specific details of each athlete’s mobilities.

### Data Collection

This study was funded by the Danish Ministry of Culture and was carried out in accordance with the recommendation of Danish Data Protection Agency. All participants gave written informed consent prior to the interview. The life story approach typically involves a series of interviews with each participant, allowing for flexibility to gain a rich and holistic understanding of participants’ experiences (Atkinson, 1998; Liversage, 2009). Participants of this study were interviewed in a series of two or three 2-h interview sessions, depending on athletes’ availability, and the richness of their stories. The individual interviews were conducted in different countries and locations, such as local cafes and hotel business centers, participants’ homes and at their work places, and university meeting rooms.

In our preparation for the interviews, we followed basic interview guidelines proposed by Atkinson (1998), including preparing background information on the athletes’ lives and generating questions for the person to be interviewed. The main purpose of contextual preparation for the life story interview is to be able to guide participants to a deeper understanding of their own experiences. As shown by Schultheiss et al. (2011), within the relational cultural framework life stories, co-constructed in the process of telling and being heard, provide a means for making meaning out of work-life experiences of migrants and how these experiences shape their lives.

A semi-structured interview guide was developed to provide a chronological framework for the inquiry. However, since historical reconstruction was not our primary concern, it was only used as a flexible, supportive tool and athletes were encouraged to follow their own preferred order in telling their stories. At the beginning, we asked participants to tell their career story in ways that were meaningful to them. The probing questions aimed to trigger memories from childhood, family, schools, and sports. From there, stories shifted into youth and adulthood, often focusing on career development in sport, education, kinship, and various transitions in athletes’ lives. In the second interview, the participant was given a chronological map that was drawn based on the first interview. This was used as an invitation to expand and further reflect on major turning points and themes identified in the first interview in order to glean additional insights into how athletes saw themselves at various points in their lives and wanted others to see them. Throughout the interviews, we were probing into shifting discourses of sport, culture, and gender, and the ways in which transnational athletes negotiated their life fit into different cultural contexts.

### Data Analysis

While emphasizing empirical data as the basis for generating knowledge, we recognize that a researcher’s *priori* conceptual understandings trickle down into the production of knowledge. To avoid diminishing the participants’ knowing by imposing conceptual categories on their experiences, we utilized abductive reasoning in our reflexive interaction with the data (Atkinson and Delamont, 2005; Sparkes and Smith, 2014). In our understanding, abductive reasoning involves a creative process of interpretation when applying conceptual frameworks adopted in this study while also acknowledging that the analytic processes were ongoing in giving meanings to first impressions as well as final product. The first step in our engagement with the transcripts was a thematic narrative analysis (Riessman, 2008; Smith and Sparkes, 2012) to map a shifting content of the cultural transition from a temporal perspective. In thematic narrative analysis, the focus is on the *whats* of the stories (content of the speech), seeking to identify common elements across participants’ experiences. The periods before and immediately following relocation emerged as common themes across the participants’ stories. The third theme was linked to prolonged engagement with a sociocultural context and was common for athletes in a long-term migration. The identified themes were related to participants’ chronological maps as transitional phases. Drawing on relevant literature, we labeled them pre-transition, acute cultural adaptation, and sociocultural adaptation.

In the second step, we worked with both structural and performative analyses (Riessman, 2008; Bamberg, 2012). In the holistic narrative analysis of structure, we sought to identify the plot of each individual life story and the central storyline that organized each athlete’s experience of cultural transitions. This approach was integrated with performative analysis to better understand *how* the process of cultural transition was enacted. According to Riessman (2008, p. 105), a performative analysis “interrogates how talk among speakers is interactively (dialogically) produced and performed as narrative.” In the performative analysis, our focus shifted to examining how transition was constructed in the athletes’ telling of their stories, including emotional responses, and the performative elements that formed psychological continuity and change in the flow of a life narrative. This involved considering the agency of transnational athletes and the transitory social field—that is, how participants positioned themselves in relation to various cultural discourses, gendered life scripts, and exemplary sport narratives—as well as gleaning insights from the reciprocal relationship between the teller and the intended audiences (Riessman, 2002; Smith and Sparkes, 2009). We identified three common performative processes that penetrated through multiple layers of unique storylines—repositioning in social networks, negotiation of daily practices and meaning reconstruction. Serving the adaptive functions, these processes were mobilized around the key developmental tasks at each phase of the transition and appear to be the underlying mechanisms through which cultural transition was enacted.

## Results and Discussion

The purpose of this study was to understand the dynamics of cultural transition and the ways in which time shapes the transition process. Our specific aims were to identify the developmental tasks and underlying psychological mechanisms, set in motion by the cultural transition, which facilitated career adaptability.

Consistent with previous research on athletic migrants, the cultural transition process was constituted in social practices and shifting modes of participation within and between sport/work and other contexts, such as school and private life. Changes in daily practices occurred both on and off the sporting field as transnational athletes experienced rupture of meanings (Hall, 1990) and social networks (Smith and Guarnizo, 2006). However, they also developed new social relations in their country of settlement while maintaining public and private engagement in their countries of origin and/or previous settlement.

Despite intensified cultural homogenization of globalized elite sport, in which a transfer to a new team/club may be viewed as a within-culture transition due to athletes’ expertise in the given sport, experiences of the athletic transition rather than transition to a new society dominated participants’ stories. On one hand, there seems to be a taken-for-granted belief in the universal language of sport, as articulated by athlete 1: “Even if there are different cultures, the hockey language is the same, like, on the ice it doesn’t matter where you come from…on the ice everything is just hockey.” On the other hand, when athlete 1 was asked to describe how he had experienced his cultural transition to the USA, he stated:

Of course, they have a different style of hockey, ‘cause here the ice rink is a little bit bigger than in the States…so it means that there is less time to do something with the puck, a lot more hitting, a little bit faster game so it took a while to get used to that hockey over there. But I think everything else just went…‘cause I was there for hockey and of course at the same time I was learning hockey, playing hockey, and outside of hockey learning to do the things how they are done in the States.

This player also reflected on how “hockey is such a big business in the States so it’s not just sport” and “there is much more bigger gap between the team and the coach,” concluding that “here, of course, coach is the coach but you can talk to the coach more easily in Finland than in the States. The coach is more kind of the big boss there.” In their unique ways, athletes were candid about differences in ‘doing sport’ and the local nuances in organizing knowledge and living routines for athletes, which points to the culturally intertwined underpinnings of the transnational athletic transition due to its embeddedness in social relations and practices.

**FIGURE 1:**
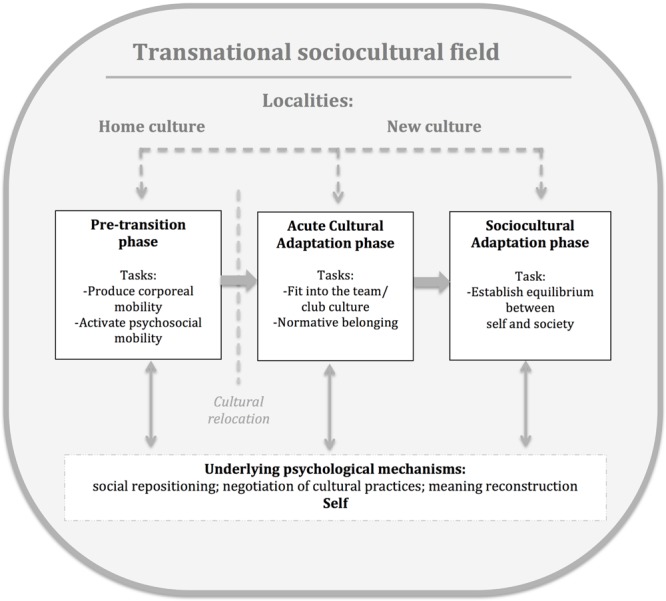
**Cultural Transition Model**.

The organizational field of sports, by means of social networks and logistical support, mediated the athletes’ cultural transition process into a new society in other domains, such as public health, school, and housing. This was in contrast to the experiences of athletes who migrated for non-sport related reasons. Therefore, stemming from the data, the Cultural Transition Model (**Figure 1**) represents the developmental tasks of a cultural transition constructed in and through work-based relationships and discursive career practices in elite sport.

In this section, we first present an empirically derived model of transition. After that we provide a thick description of the cultural transition’s phases and discuss the athletes’ accounts of living through that process.

### Cultural Transition Model

The model in **Figure 1** shows that the cultural transition process consists of three phases, two of which occur post relocation. While the temporal phases were extracted from the data by means of analytical abstraction and are presented in a sequential progression, they should not be interpreted as unfolding in a simplistic linear manner. We suggest that the transition process is relational, meaning that it does not merely unfold, but is rather constructed within a transnational sociocultural field dynamically and subjectively adjusted by individuals to the multiplicity of cultural contacts in various localities. The psychological domain is embedded in the fluid trans-local cultural field of social practices that constitute and sustain daily functioning and relational experiences of athletic migrants. Although individual experiences are unique and “cultures play infinite variations during the course of development and daily activity” (Berry, 2009, p. 364), our data analyses suggest that the athletes interviewed possessed common developmental tasks that mobilized the adaptive strategies/mechanisms in cultural transition. As **Figure 1** demonstrates, we propose three underlying psychological mechanisms through which cultural transitions are enacted. These basic processes are adaptive responses that might be hidden beneath an array of culturally patterned behaviors and discourses that, in turn, may or may not lead to a successful task resolution in a particular cultural context. It seems important to reiterate that we identify the underlying psychological mechanisms as adaptive because they are mobilized to regulate one’s mode of being in cultural transition.

### Phases of Cultural Transition

Athletes’ narrations encompassed various temporal and spatial vantage points—reflecting on their past experiences, they would make connections with their lives today and expectations for the future. The ways in which narrators navigated between ‘here’ and ‘there’ also highlighted the fluidity of their adapt-abilities to modify affective responses, interpretations and behaviors in various locations.

In the following description of the cultural transition process, we have attempted to add a nuanced analytical depth to our representation of the ways in which athletes’ (a) repositioning and calibration of social relations and networks, (b) negotiation of living cultural practices, and (c) decoding and reconstruction of meanings animated the psychological continuity and change at each phase. It is important to note that the aforementioned adaptive mechanisms had a varied degree of experiential saturation along the transition timespan, all shaping, nevertheless, the psychological functioning of the self in cultural transition.

#### Pre-transition

This phase became visible in athletes’ stories as they talked about the time when they had been contemplating a transnational migration. In professional and high revenue college sports, gathering basic information about teams, coaches, and locale through various social networks (e.g., intermediaries and friends of friends) appears to be a common practice as attested by athlete 4, “I think everybody that go [migrate], have talked to somebody that’d been there.” Reflecting on his decision-making to sign abroad, pro hockey player 15 recalled, “since 2000 there had been many Finns who went to play in Sweden, so there was the culture of going to Sweden.” Transnational athletes do the groundwork of learning about coaches and team practices, fans and club culture, living and groceries, so that “lots of guys know beforehand what to expect” (athlete 4, ice hockey).

Often players get a chance to meet coaches and teams either virtually (e.g., Skype/conference calls) or to visit the place before relocation for try-outs, which assists them in making a more informed decision about mobilizing their athletic career and life course in general. For example, it was important for athlete 11 to develop her game by playing on a highly technical football/soccer team. Selecting a team that was coached by a Latin American coach known for his meticulous attention to footwork dominated the player’s decision-making when accepting an athletic scholarship in an American university. Moreover, several Skype sessions with the coach reassured her of the right fit within the team and school. The constitutive dynamics of agency and networks in animating a potentially successful transnational career transition was further exemplified by a young African footballer:

I wanted to play in Portugal, but the coach when I went [to Portugal for try-outs] couldn’t speak English. He spoke Portuguese to me. I don’t understand Portuguese…so I don’t understand what he’s saying. Then I called my agent and said I want to leave…That’s why I came to [Scandinavian country] to play. They said that the coach is used to speaking English.

In addition to being able to produce transnational mobility, the crucial task of a pre-transitional phase is to activate psychosocial mobility that is necessary for navigating diverse meaning systems and negotiating cultural practices. Although not many athletes talked about crossing the symbolic boundaries of various cultural discourses, most of them looked for possibilities to experience something new and were open-minded about the diversity of social and cultural forms of being-in-the-world. This was effectively summarized by athlete 4 (ice hockey):

You need to have an open mind that things are going to be different. Different countries have different kind of cultures, so you have to be ready for the different personalities or different ways of living. Those are big things, I think, to adapt to.

Importantly, by tapping into transnational networks and learning about a new cultural site, athletes initiated a process of psychological disengagement with familiar people and places while developing a relationship with the new locale. As observed by Ronkainen et al. (2016) in the study of transmigrant runners’ negotiations of their serious leisure practices in migration to China, the Western skilled migrants planned to stay there for a limited period of time and viewed their challenging experiences as temporarily. Similarly, despite considering transnational mobility either as an opportunity for upward mobility or a necessity, the participants in the present study constructed migration as temporal before returning home.

For married or cohabited participants, it was also the period of making decisions whether or not their partners and children would migrate with them. While the traditional gendered life-script was taken for granted by some athletes (e.g., “for me it felt very normal that she [girlfriend] would come with me,” athlete 1), the need for negotiating alternative ways of keeping romantic and family ties alive was voiced by several athletes, who narrated reconstructing meanings of canonical scripts. For example, professional athlete 4 faced a dilemma whether to sign an overseas contract although his wife wanted to stay at home for her child’s perceived benefit. Despite his relatives’ intense disapproval of the wife because “she knew what she was marrying into,” the athlete started narratively repairing the biographical rupture caused by transnational migration. First of all, they “were okay because [he] defended her” and then when he signed the contract, he continued negotiating family life in migration as the following quote illustrates:

I feel guilty – in a way I love what I do. I chose to do this as a living […] but I still have guilt for not being there for the kids all the time. I guess the comfort in knowing that my wife is doing such a good job helps me a lot to cope with everything. I know she has my back.

To summarize, the pre-transition phase is indispensable in physically and mentally preparing for the challenges in undertaking a cultural transition, which often means searching for opportunities as well as decoding and reconstructing established life scripts and career narratives. In this sample, poor or incorrect expectancy of the cultural differences which awaited the athletic transmigrants in their host environment and neglecting to expand on normalized ways of being were linked to confusion, resentment, emotional disconnection, and social withdrawal at the subsequent stage. The mobilization of sporting trans-local networks was the key for engendering mobility as part of the transnational career development. Moreover, since geographical mobility puts a strain on family life (Gmelch and San Antonio, 2001; Roderick, 2012) and potentially can prompt family disruption and conflict, the pre-transition phase may provide the athlete and their families with time to negotiate necessary adjustments toward work-family balance.

#### Acute Cultural Adaptation

For many athletes, the acuteness of this phase was lived and felt through loneliness and attempts to fit in with the cultural patterns of group life. Athlete 9 (football/soccer), who was interviewed 8 months following migration, immediately evoked these themes by recalling, “when I came, it was very difficult for me. I’m just lonely. I just live alone. I don’t have friends. I don’t have any place to go.” He also struggled with the cold weather and hard training, trying simultaneously to make sense of “running in the pitch” without the ball, “we just use the cones, so it’s very different [from home].” His coach was not happy but understanding, “he just said I should look. I would just sit looking but not train because of the weather.” The footballer confessed, “I play better when I see the sun.” This athlete’s experiences were concurred by others, who missed family and friends as well as familiar spaces, landscapes, and ethnoscapes, attesting further to the felt rupture of their daily life, inner meaning, and established routines.

The symbolic structure of the meaning domain became visible in feelings of loneliness amidst people and alienating experiences as exemplified by a mixed race Scandinavian athlete, who was positioned simultaneously as ‘other’ and ‘black’ in the USA. English was not her first language and when she moved to live with her extended family, “everybody made fun of [her] accent…but then, in school people were saying, ‘you talk like a black person.”’ Her response was, “how does a black person talk? What does that mean?” Reliving her poignant story, the athlete kept raising that question throughout her narration:

I remember when I was walking to school with my oldest cousin—the first day of school—she’s like ‘all the boys are going to like you ‘cause you’re light-skinned and you have a big butt.’ And I was like, ‘what does that mean?’ I didn’t understand, and she was like, ‘you also have good hair.’ I didn’t understand what she meant by that because in [Scandinavia], well, in [Scandinavia] having a big butt isn’t necessarily a good thing.

Having lived the trajectories of meaning embedded in a particular sociocultural context, most athletes had to learn the ways of navigating, negotiating, and evolving their own understandings within cultural patterns of a new site. Those who struggled to repair the mismatch between their own mode of being and social context (e.g., due to culture distance, marginalization, and exclusion) were likely to feel the loss of self as exemplified by athlete 12, “I’m just lost in translation. I don’t know who I am.”

The highly dynamic reconstruction of meaning and social repositioning intricately worked together with the negotiation of daily practices on and off the field. Religious athletes, for example, first sought a church or congregation to which they belong, but often had to come to terms with inability to attend worship (e.g., Sunday games) or to relate to ceremonies because they were conducted in a language they did not understand. Albeit investing time and energy to orienting themselves in a new cultural locality, it was the sporting social context that was of most importance to transitioning athletes as they recounted learning and trying to understand the team norms and practices: “I was quiet and I was watching. I think I was, in my way, trying to learn the other players by watching them – or by watching how they act” (athlete 6, basketball). Responding to the question of what was his strategy to facilitate entry into the team, athlete 15 (ice hockey) shared, “stay open, talk to the guys, just be normal and do what they do.” Player 9 (football) agreed, at first “it was so odd for me, very hard. I didn’t understand the training so I would just look at my teammates…now I have the experience. I understand the training. I understand what I’m doing now…I play the way they play.”

Whilst some behaviors were not difficult to compromise in order to feel part of the team—“I laughed at a lot of stupid jokes” (athlete 4), other practices were deeply ingrained in cultural meanings and symbols associated with age, class and prestige, to name a few, creating inner conflict and external misapprehension. For a young African footballer, who ‘made it in Europe,’ it was incomprehensible to accept a bicycle as a means of transportation because “bike is for kids.” When the interviewer reassured him that it is okay to use a bike, the athlete responded:

Yeah, that’s what they [in the club] told me. But I said, ‘it’s not okay.’ They said, ‘it is okay.’ I see a lot of people using a bike. Even the coach, you know, sometimes uses his bike. So I said ‘maybe I will do that,’ but I don’t know the day.

Explaining what bicycle meant for him, the footballer was shifting his subject position between ‘here’ (e.g., my teammates use the bike) and ‘there’ (e.g., ahhh, look at [his name]!) in an effort to reconcile his uneasiness and tension: “I don’t care if they see me. I don’t care if they…so I will just bike, but I don’t know the day.”

As already indicated, sport social networks mediated the transnational athletes’ cultural transition into a new society by providing logistic support (e.g., visa, housing, healthcare, and taxes) and acting as cultural guides. High-revenue sports clubs and teams had resources to offer formal support and hired help, such as interpreters, drivers, and ethnic cooks, in return for immediate results, as suggested by a pro hockey player, “they expect big things from imports…if you’re an import from outside, I don’t think they are very patient.” A female orienteer on a pro contract for the competitive season echoed, “there were very many foreigners there, so it was competitive. You had to have these good results, otherwise they would just kick you out from the club.” Athletes from less commercial sports relied more on personal networks, coaches and teammates. This was evident in the story of athlete 3 (dance), who shared the cultural background with one of his coaches, as he stated:

She had been living here for a long time so she, when I needed something or didn’t know how things work here, I could always ask her and she always helped me with those things. So I had a feeling of security that I’m not left alone to deal with it.

The team/club’s culture was intertwined with athletes’ perception of whether their psychological needs were met and how well their own goals and values were fitting in the group patterns, which in turn impacted their performance as well as motivation to stay and adapt to social life in the country. For example, while a Scandinavian soccer player in the USA felt that “it was very easy for [her] to go there because it’s exactly the same way that it is here [home country],” she credited her Brazilian coach for subtly creating a cultural space that facilitated learning and co-construction of shared experiences and norms. Similar to previous cultural transition research (e.g., Ryba et al., 2012; Schinke et al., 2013), this athlete reflected how her teammates formed a safety net of belonging that enabled her athletic development while simultaneously providing pre-constituted ‘experiencings’ of social life outside soccer. In contrast, the aforementioned orienteer (athlete 12) was “kicked out” for underperforming and was relieved to return to the country of previous settlement in which orienteering “is on high level but also fun” and “nobody expects you to win all the competitions.” Comparing her experiences in the two teams in two Nordic countries, the participant attested to the constitutive relationship between sociocultural processes and individual processes:

In Denmark, they took me as a person. In Finland, they took me only as an athlete. It’s very different. So, in Finland, I kept my relationship at the very basic level—I do what I’m asked, but that’s it. But in Denmark was friendship, so I wanted to do it—to get along with others and do well. I didn’t talk about that to the team leaders [in Finland] because I was already thinking that they don’t care about runners. They only care about your results.

This experience made her realize that although her lifestyle was that of a professional athlete, she was more content in mobilizing her life course through a semi-professional athletic career and work to support herself and her athletic pursuits. The provided examples also reveal the ways in which psychological responses to the cultural transition’s tasks are embedded within relational contexts; thus, mutually constituting a career pattern to fit sport/work into the athlete’s life design (Richardson, 1993; Savickas et al., 2009).

To summarize, migration experiences involve intense feelings of loss and loneliness on the one hand, but also excitement and hope of new opportunities on the other. Stemming from Kitayama et al.’s (2007) presupposition that a self-system provides a culturally grounded principle for spontaneous mobilization of thoughts, feelings, and actions, acute adaptation is characterized by a dynamic psychological work of calibrating adaptive responses to new social contexts. The decoding and reconstruction of meanings is central in the process of self-transformation and is stimulated by social repositioning and negotiation of cultural practices in daily living. Although emotional intensity of the acute phase is temporal, it may last for several months and encroach into the subsequent adaptive phase. Athletes unable to establish an affective and cognitive connection with the new locality by integrating new cultural meanings, and consequently expanding their own mode of being, were dissatisfied with their professional development and had to look for new possibilities of constructing the life-course.

#### Sociocultural Adaptation

In contrast to intermittent mobilities, the sociocultural adaptation phase is associated with the migration that has connotations of permanency or long-term stay (Koser and Salt, 1997). For example, athlete 10 (alpine skiing) relocated to Sweden for training as she felt that female athletes were not given same opportunities as boys and men in her home country. Although the athlete did not articulate any emigration intent at the interview, she had been living in Sweden for several years and was planning to combine her semi-professional athletic career with further education in an athlete friendly Swedish university. In our study of the transnational athletes’ dual careers (Ryba et al., 2015b), we have demonstrated that despite agency playing a central role in the ways through which athletes navigate social structures to bring authenticity and meaning to their life in dual career pursuits, the discursive and material conditions in different locations in which athletes are situated nevertheless enable or constrain mobility (see also Carter, 2011).

Athletes in this sample, who expressed intent to stay in the country of settlement, communicated their content with *both* their athletic environment and society at large. These participants seemed to feel congruency of their own values and lifestyles with the local cultural norms animated through daily discourses and practices in various social contexts. In line with the findings of a recent satisfaction study of mobile employees on international assignments (Tretyakevich et al., 2015), athletes who considered themselves psychologically adjusted to the new culture, reported higher levels of satisfaction with non-sport related aspects of everyday life. As exemplified by pro hockey player 4:

…where I grew up and Finland, culturally, it’s very similar… our health care is the same as here, our taxes are the same as here, we have lots of similarities, it’s incredible. I felt – yeah, that’s why I fit in so well here with my cultural upbringing, here rather than elsewhere.

Furthermore, this and other participants were cognizant of how sport and non-sport contexts were interdependent as well as interlinked with the self or agency in producing adaptability of their careers. Most of them took advantage of their simultaneous embeddedness in several locations to change clubs or teams in pursuit of a better fit. The argument presented by Savickas (2005) that career is enacted by expressing self concepts in relational contexts was infused with life by athlete 4 as he attested:

I rely on my hard work, and being team player, that’s what I love about here. There’s no BS and it’s straightforward—that’s not everywhere in Finland, but here, that’s the culture that’s here. As an older player, I think that’s an important thing to have, a place you can admire the style and the kind of people that reflects who you are and how you were brought up.

Albeit considering themselves well adjusted at this phase, the participants also exhibited variations in their acculturation and life-theme motives, which in part directed their performative talks and actions. Many athletes had plans to return home after a fixed-term contract, graduation from a university or athletic retirement, and therefore had mixed feelings about their belongingness, often exacerbated by the fact that their families were not permanently living with them. For example, while athlete 15 liked Sweden—“I actually learned to like Sweden a lot as a country and even nowadays I still go to read some news on internet just to see what’s going on in Sweden”—and understood the local language to function competently and autonomously in daily social situations (e.g., housing and taxes), most of his time outside hockey was spent online in Skype sessions with his wife and children as he confessed, “Sweden was a temporary place…I guess I just missed things outside ice hockey.” For this athlete, a sense of home was strongly connected to a physical place and the people in this specific locale. Therefore, after 2 years in Sweden, he returned home to maintain a professional hockey career as well as sustainability in his personal life.

Previous research on labor migration indicates that it is precisely the athletic migrants’ transnational and multiple embeddedness that serves as a catalyst of their mobility (Engh and Agergaard, 2013), facilitating also career continuity. The transnational athletes’ active management of their careers appeared to be linked to affective and normative commitment to various relational contexts inasmuch as most of them had established a sense of transnational belonging—feeling at home in multiple localities through transcultural forms of social engagement. Yet developing transnational identity was a process of challenging the self, as asserted by a Southern European basketball player (athlete 7):

It was challenging to come to Denmark, first. Then it was challenging to go to [a Nordic country], definitely. Then it was challenging going to the USA. It was so far and it was just a completely different culture—then going to [another American state], it was challenging. The first year. And then maybe the second and third year it was better, but then coming [back] to Denmark was challenging. So it was all challenging. I mean, I’ve been challenging myself all the time…just being outside of my comfort zone, in a way, it was everything—language, culture, people.

The gradual molding of self, as illustrated by athlete 7, forefronts the idea that psychological processes are emergent outcomes of interaction with experience derived in a myriad of social practices in a given cultural locality (Vygotsky and Luria, 1930/1993; Markus and Kitayama, 2010). The transnational athletes’ acquired abilities to continuously re-position themselves in transient contexts, calibrating their social relations and practices while navigating shifting meanings, further highlight the constitutive link between the developmental tasks of cultural transition and career adaptability. That is, the work of transition is a psychological repairing of the cultural rupture of daily life that results in a more expansive and fluid psychological self-system. The underlying mechanisms of transition identified in this research (see **Figure 1**) were uniquely mobilized in response to the tasks at each phase of cultural transition furthering growth through work and relationships in shifting sociocultural landscapes.

## Limitations of the Study

In this article, we did not explore issues surrounding gender and sport sub-culture in mobilization of the transnational athletic career. Our focus was on sport-based cultural transitions of athletes who had a various degree of relocation support through sports organizations and athletic clubs. Therefore, it is reasonable to anticipate a different set of challenges facing athletes in unassisted cultural transitions, such as restricted athletic team/club membership and limited access to training facilities at the host site. Moreover, although some participants felt they had to migrate in order to secure their athletic careers, the athletes in this study can be characterized as skilled migrants and had more agency in negotiating their relocations and sport contracts. Hence it is important to consider power relations and immigration policies between the dominant culture and transnational migrants when applying the proposed framework to cultural mobility issues, especially in career counseling with marginalized and dislocated groups. While we believe that the Cultural Transition Model is applicable across different sports and potentially can be appropriate for developing psychological support of skilled migrants and their families, further research is needed to test the model in different contexts.

## Practical Implications

The athletic migrants’ life stories clearly suggest that cultural transition has important implications for the direction of their careers and also their opportunities to have a meaningful life. Their transnational career accounts revealed a lack of psychosocial support available for transitioning athletes in sport organizations and, therefore, call for the need to make career and life design counseling available for them. The findings of this research offer some important insights for sport psychologists and career counselors regarding the ways in which they can assist their clients with the psychological work of mending cultural rupture. We believe that these recommendations can, moreover, be applicable in counseling with other groups of high-skilled workers who pursue work-related migration.

Firstly, the temporal model of cultural transition presents a succinct overview of the transition process, including the developmental tasks that change with time. The theme of time served as an important but highly subjective context in athletes’ experiences of cultural transition and should therefore be approached from an idiosyncratic felt-sense framework in individual counseling. While there are also large variations in a culturally contingent repertoire of attitudes and behaviors through which individuals engage with the transition process, we propose that the underlying mechanisms of cultural transition discussed above are common to adaptively sustain human functioning.

Secondly, our findings highlight the importance of activating the psychosocial mobility pre-transition. As Bimrose and McNair (2011, p. 330) argued, “migrants represent a client group for whom adaptability and identity are crucially the key tasks for career intervention”; from a narrative perspective, career counseling with athletes prior to transnational migration could involve helping them to become aware of their established career narratives and life scripts and the potential need to reconstruct these narratives in cultural transitions.

Thirdly, transmigrants often move alone and might experience intense loneliness, especially at the acute cultural transition phase. As Stuewe-Portnoff (1988, p. 548) asserted, relational connection with people requires not only a physical proximity, but also a shared meaning domain wherein “the world means the same thing to others that it means to me.” Our findings suggest that receiving organizations assessed rather poorly the extent of how cultural rupture of daily life impacted athletes’ wellbeing. Organizational and sport psychologists working in teams could increase staff awareness of the challenges faced by transnational athletes and the ways of supporting them through transition, thereby decreasing the ‘acculturation load’ faced by incoming athletes.

Fourthly, athletic transmigrants’ economic success and social status does not depend exclusively on rapid acculturation and assimilation into the host society that opens the possibilities for new, still unexplored adaptation pathways. It is important for career counselors to recognize that there are numerous ways to incarnate a transnational career, which may contain multiple intermittent mobilities and/or transnational migrations. While simultaneous embeddedness in several localities and cultivation of social networks are integral for maintaining transmigrants’ mobility, certain career transitions may be decisively transient. Consequently, as our findings indicate, athletic migrants may rather invest time and energy into learning English than a (marginal) local language and may be reluctant to embrace culture of another society while, nevertheless, being eager to adapt to it instrumentally.

Fifthly, for migrants actively engaged in transnational networks and activities, transnational belonging is essential for the formation of identity and self-concepts. In line with previous transnational studies (e.g., Colic-Peisker, 2010; Ryba et al., 2015a), the findings of this study indicate that highly skilled migrants identify strongly with professional competencies. Therefore, their career–life identities may derive meaning from a culturally hybrid transnational field rather than the country of origin. Extrapolating from Blustein’s (2011) proposition to consider culture as a form of holding environment that serves adaptive functions, the transnational community may serve as a vital holding environment for migrant workers as they cope with cultural transitions.

## Conclusion

The results of this study enrich the International Labour Organization’s (International Labour Organization [ILO], 1999, p. 7) concept of decent work by grounding the ILO’s themes of “working conditions,” “balancing work and family life,” “equal recognition,” and “enabling women to take control over their lives” in the psychological dimensions of work-based movement and transnational mobility. Drawing on a transactional approach, which views social-historical influences as constitutive at the level of individual experience, our research findings provide a compelling support for the sociocultural constitution of transition and relational construction of career adapt-abilities. Given the limited opportunities for long-term contracts, fierce competition and global flow of talent in the sport industry, today’s athletes are often put in compromising situations where mobility is required at the expense of stable social networks, local belonging and family life. As the study indicated, host organizations were often prepared to assist in practical arrangements, but less likely to offer psychological support in adaptation to cultural transition. Hence it is important to consider whether individual difficulties in the cultural transition to specific organizations are a personal failure or an indication of organizational problems. This claim resonates with previous arguments that, although in the new economy individuals must take responsibility for their own career development, there are also highly interactive mutual dynamics between organizational and individual career development, “in which both parties are at once the agent and the target of career influence” (Lips-Wiersma and Hall, 2007, p. 771).

## Author Contributions

TR developed the study concept. TR and NS designed the study. TR and NR conducted the interviews and data analyses. TR and NS developed the cultural transition model. TR wrote the manuscript and received substantial input from both co-authors. All authors approved the final version of the manuscript for submission.

## Conflict of Interest Statement

The authors declare that the research was conducted in the absence of any commercial or financial relationships that could be construed as a potential conflict of interest.
